# Estimation of cup orientation on intraoperative fluoroscopy in total hip arthroplasty: how safe is the safe zone—an experimental study

**DOI:** 10.1007/s00402-025-06025-1

**Published:** 2025-09-08

**Authors:** Roman Maduz, Michel Schläppi, Peter Wahl, Emanuel Benninger, Christoph Meier

**Affiliations:** 1 Present Address: Division of Orthopaedics and Traumatology, Cantonal Hospital Winterthur, Winterthur, Switzerland; 2 Orthopaedic Clinic Rychenberg, Winterthur, Switzerland; 3 Department of Biomedical Engineering, University of Basel, Allschwil, Switzerland; 4 ARTORG Centre for Biomedical Engineering Research, Faculty of Medicine, University of Bern, Bern, Switzerland

**Keywords:** Inclination, Anteversion, Fluoroscopy, Total hip arthroplasty, Acetabular cup orientation, Safe zone

## Abstract

**Background:**

Accurate acetabular cup orientation in total hip arthroplasty (THA) is crucial for successful outcomes. Intraoperative fluoroscopy may be used to evaluate acetabular cup placement. This study aimed to evaluate the accuracy of purely visual estimation of cup inclination and anteversion using intraoperative fluoroscopy, considering different surgeon experience levels and cup designs.

**Methods:**

Thirty-five surgeons with varying levels of experience participated in the study. Standardized fluoroscopic images depicting two different cementless acetabular cup designs placed in a bone model were used. Inclination values ranged from 20 to 60°, while anteversion values ranged from 0 to 40°, both in 5° increments, resulting in 162 combinations of cup orientation. Each participant received a randomly compiled sequence of all images and was provided with instructions for estimating inclination and anteversion angles, and was asked additionally to categorize cup orientations into predefined safe zones, utilizing two definitions: (a) an institutional safe zone (inclination 35 to 45°, anteversion 10 to 20°), and (b) the safe zone according to Lewinnek et al. (inclination 30 to 50°, anteversion 5 to 25°). Participants had no time limit and were not allowed to use measuring tools during the estimation process.

**Results:**

No significant difference in the precise estimation of inclination and anteversion was found among surgeons of varying experience levels. However, the ability to correctly identify whether cup orientation fell within predefined safe zones improved with surgical experience and seniority. Cup design influenced estimation of inclination, with one design showing superior accuracy. Estimation of anteversion remained consistent across designs. The influence of anteversion on inclination estimation and vice versa was minimal. Safe zone definitions did not significantly affect classification accuracy between cup designs.

**Conclusion:**

While surgical experience did not improve angle estimation accuracy in degrees, it was associated with more accurate identifications of positions within clinically relevant safe zones. Cup design influenced inclination estimation. Our findings emphasize the importance of precise cup positioning in THA and highlight areas for potential improvement in surgical practice and training.

## Introduction

Ensuring successful function and longevity of total hip arthroplasty (THA) hinges significantly on the correct orientation of the acetabular cup. Postoperative instability and dislocation remain two of the major issues that are associated with cup malposition [1–3]. Furthermore, acetabular cup malposition has been linked to restricted range of motion, component impingement, increased wear, and development of periprosthetic osteolysis [4, 5].

While some surgeons rely on intraoperative anatomy and landmarks only, without any intraoperative imaging for confirmation of proper acetabular cup placement, others employ advanced navigation techniques, specific guiding tools, or robotic-arm assisted technology [6–9]. Intraoperative fluoroscopy has been widely used, particularly for the anterior approach in supine position [10–12]. Despite its widespread use, intraoperative fluoroscopy presents challenges that may compromise the accuracy of assessment of the orientation of the cup. Factors such as image quality, equipment interference, variations in pelvic tilt compared to preoperative planning, and improper image intensifier alignment have been identified as potential limitations [13–15].

Although fluoroscopy is commonly used to guide acetabular cup positioning, there is limited evidence systematically evaluating the accuracy of visual estimation of inclination and anteversion based on fluoroscopic images.

The primary objective of this study was to evaluate the accuracy of visually estimating cup inclination and anteversion using fluoroscopy, as done intraoperatively, without any additional measuring instruments. We also aimed to investigate the influence of surgical experience and specific cup designs on the estimation accuracy. Furthermore, we analyzed how different definitions of safe zones influence the perceived accuracy of cup positioning to get relevant conclusions to clinical practice.

## Methods

### Reference images

**Fig. 1:**
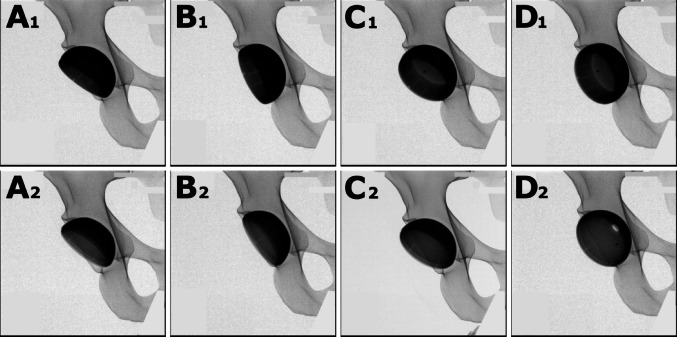
A selection of fluoroscopy images as they were presented to the participants to estimate inclination and anteversion. In the upper row, cup Design 1 (Versafit) and in the lower row cup Design 2 (Allofit). The subscript number corresponds to the acetabular cup design type. Image **A** shows the ideal target cup orientation. **B** shows correct anteversion with an inclination outside the safe zone. **C** shows correct inclination with an anteversion outside the safe zone. **D** shows excessive anteversion and inclination, both outside the safe zone

Two cementless acetabular cup designs were used: Design 1 (Versafit, Medacta, Castel San Pietro, Switzerland) and Design 2 (Allofit Alloclassic, Zimmer Biomet, Zug, Switzerland). Components with nominal size 54 were implanted in a synthetic bone model of a human pelvis (Synbone, Zizers, Switzerland). In short, both cup designs differ regarding shape at the opening (Versafit having a lateral overhang resulting in a face change of the opening of 5°, whereas Allofit has a symmetrical design), shape at the pole (Allofit having a marked pole flattening to ensure equatorial pressfit, whereas Versafit has only a slight pole flattening) and thickness (Versafit being designed to accommodate ceramic inlays, it is relatively thick, partly impairing identification of the opening under fluoroscopy, whereas Allofit is designed relatively thin-walled). Radiographs of both designs are provided in Fig. 1. Radiographs were captured for all possible combinations of anteversion (0–40°) and inclination (20–60°), both in 5° increments, using a C-arm (Ziehm Vision RFD, Ziehm Imaging, Nuernberg, Germany), positioned above the synthetic pelvis, centered over the hip joint, as during THA using an anterior approach. To obtain accurate radiographs, the C-arm was oriented for a true anteroposterior view of the pelvis, with the coccyx centered 2.5 cm above the symphysis, without rotation or torsion, as described by Clohisy et al. [16]. Despite challenges in maintaining the exact position of the cup due to weakening of the interface with the synthetic bone after multiple cup impactions, inclination and anteversion angles were measured using a large digital goniometer, respectively verified on the X-ray images using the method outlined by Murray and Liaw et al. [17, 18]. Various angles of inclination and anteversion were assessed, using a straight cup impactor (perpendicular to the opening plane of the cup) as reference to define the acetabular axis [17]. Radiographic inclination was defined as the angle between the longitudinal axis of the pelvis and the projected acetabular axis on the sagittal plane [17]. Accordingly, anteversion was defined as the angle between the coronal plane and the axis of the cup. Inclination values were varied from 20 to 60° and anteversion values from 0 to 40°, both in 5° increments. All radiographs were acquired using this standardized setup and were identical for all participants.

### Evaluation and analysis

Thirty-five orthopaedic surgeons and orthopaedic residents with varying levels of experience, all affiliated with our teaching institution, were invited to participate. Individual characteristics such as experience (number of performed THA) and seniority (participant’s role within the division, including trainees, junior and senior consultants) were recorded for each participant. Prior to the commencement of the study, participants received detailed instructions. The participants were required to use a computer with a standalone screen or a laptop (no tablets or mobile devices with reduced screen dimensions were allowed). A summary of the institutional safe zone, defined as a target inclination of 35 to 45° and anteversion of 10 to 20°, was provided. Additionally, Lewinnek’s safe zone (acceptable range for inclination 30 to 50°and for anteversion range 5 to 25°) was outlined [19]. A randomly compiled sequence of pictures, containing the complete set with 81 images for each cup design, was generated for each participant using R statistical software [20]. The survey was conducted using the software Findmind [21]. Participants were not permitted to use tools such as goniometers or specific software to assess or measure cup orientation. For each image, participants were required to define first if the orientation of the cup was within the given safe zones (Lewinnek’s safe zone and the institutional safe zone), and then to evaluate the inclination and anteversion in degrees. Although the radiographs were generated in 5° intervals, participants were unaware of this and were asked to estimate the angles as continuous values, simulating real intraoperative judgement. Estimation accuracy was defined as the absolute difference between the participant’s estimated angle and the true angle for both inclination and anteversion. Participants were required to perform the assessment solely by visual analysis of the provided radiographs, one by one, without the ability to scroll back to reevaluate previously given assessments. However, no time limit was imposed on the participants. The sensitivity (accurate identification of cup orientation falling within the predefined safe zones) and specificity (correct identification of cup orientation outside these safe zones) of both safe zones and cup designs were determined through the evaluation of the images.

### Data analysis

All measurements were analyzed and visualized using R statistical software [20]. Estimated angles for cup inclination and anteversion were reported as means with standard deviation. A mixed-effects ANOVA for repeated measures was conducted to assess the differences between the deviations in inclination and anteversion angles among the two acetabular cup designs. A logistic regression was conducted to assess the impact of the physicians’ experience and acetabular cup design on the relative frequency of correctly classified images according to the safe zones. A chi-squared test was conducted to compare the numbers of correctly evaluated images within the safe zone between the two cup types. The significance level was set at 0.05. 

## Results

All 35 invited orthopaedic surgeons and residents completed the survey. Each participant rated the complete set of images of 81 different combinations of inclination and anteversion for each cup design, resulting in a dataset comprising a total of 2’835 rated pictures for each cup design. Regarding experience, 13 raters (37%) had performed between 0 and 10 THA, 15 raters (43%) had performed 11 to 50 THA, and 6 (17%) had performed 51 to 100 THA. Only one rater (3%) had performed more than 100 THA. No statistical difference was found regarding accuracy of estimation of either inclination or anteversion depending on the participants’ experience (number of performed THA and seniority). However, sensitivity and specificity for both the institutional and Lewinnek’s safe zones improved with increasing experience (number of performed THA (institutional: *p* = 0.001; Lewinnek: *p* = 0.009) and seniority (institutional: *p* < 0.001; Lewinnek: *p* < 0.001).

**Fig. 2:**
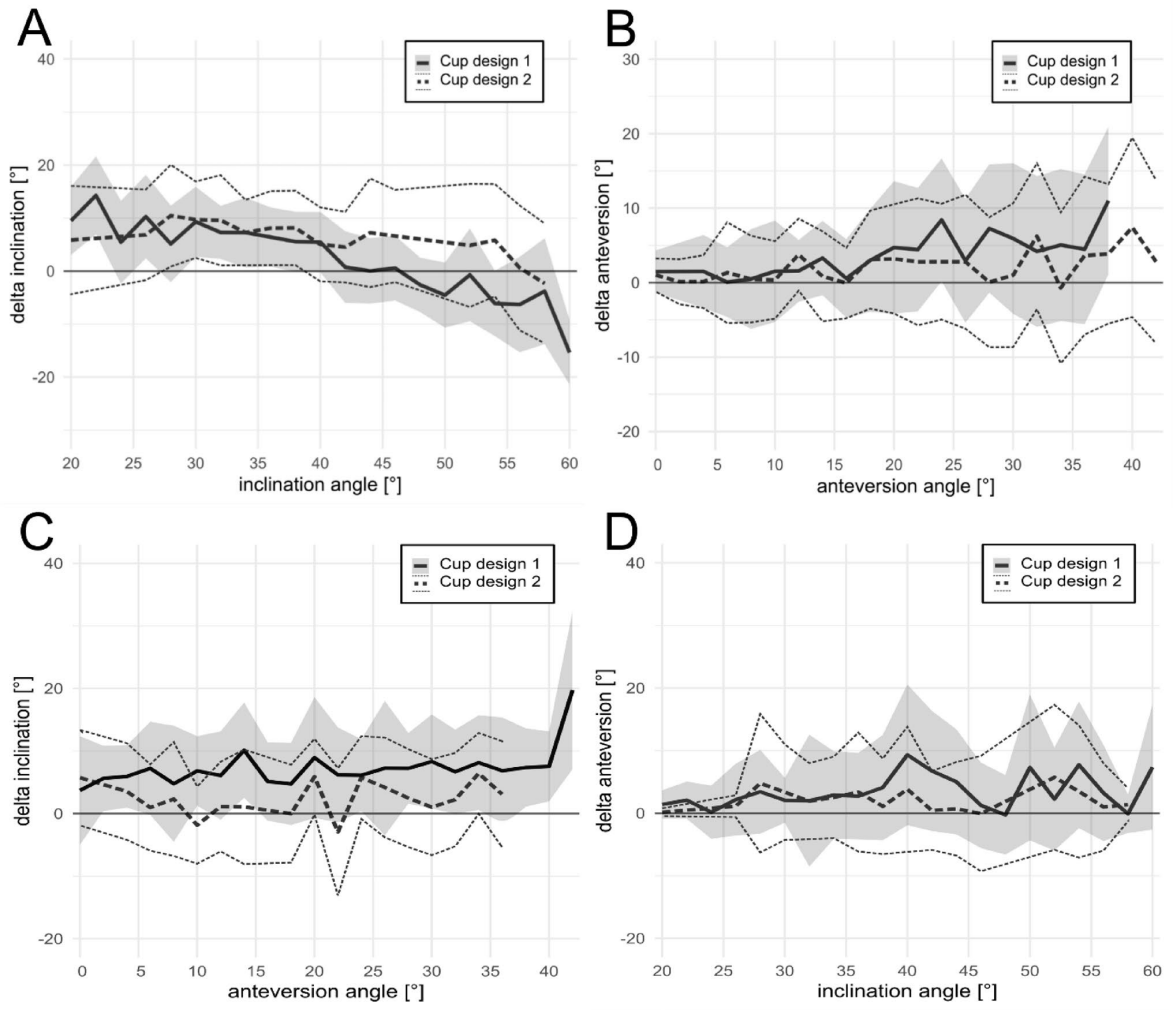
**A** Accuracy of inclination estimation, shown as the difference between the estimated and measured inclination angles, depending on inclination. **B** Accuracy of anteversion estimation, shown as the difference between the estimated and measured anteversion angles, depending on anteversion. **C** Accuracy of inclination estimation, depending on anteversion. **D** Accuracy of anteversion estimation, depending on inclination. Data are displayed as mean ± 1SD, cup design 1 in dark with continuous lines and cup design 2 with dashed lines

In our analysis, the estimation of inclination was significantly better for Design 2 compared to Design 1 (Fig. 2A/C, *p* < 0.001). While lower inclinations were generally overestimated and inclinations > 45° underestimated, a different pattern was observed for Design 2, where inclination was generally overestimated by around 5° up to 55° of inclination. Regarding anteversion, no difference was found for the two cup designs (Fig. 2B, *p* = 0.073). Overall, anteversion angles displayed a trend of overestimation as cup anteversion increased, as illustrated in Fig. 2B, with accurate estimation achieved for low anteversion angles ranging from 0 to 15°.

The anteversion angle had only a marginal impact on the quality of estimation of the inclination (*p* = 0.979, Fig. 2C). Similarly, the estimation of anteversion related to a given inclination angle showed no significant impact (*p* = 0.171, Fig. 2D).

**Fig. 3:**
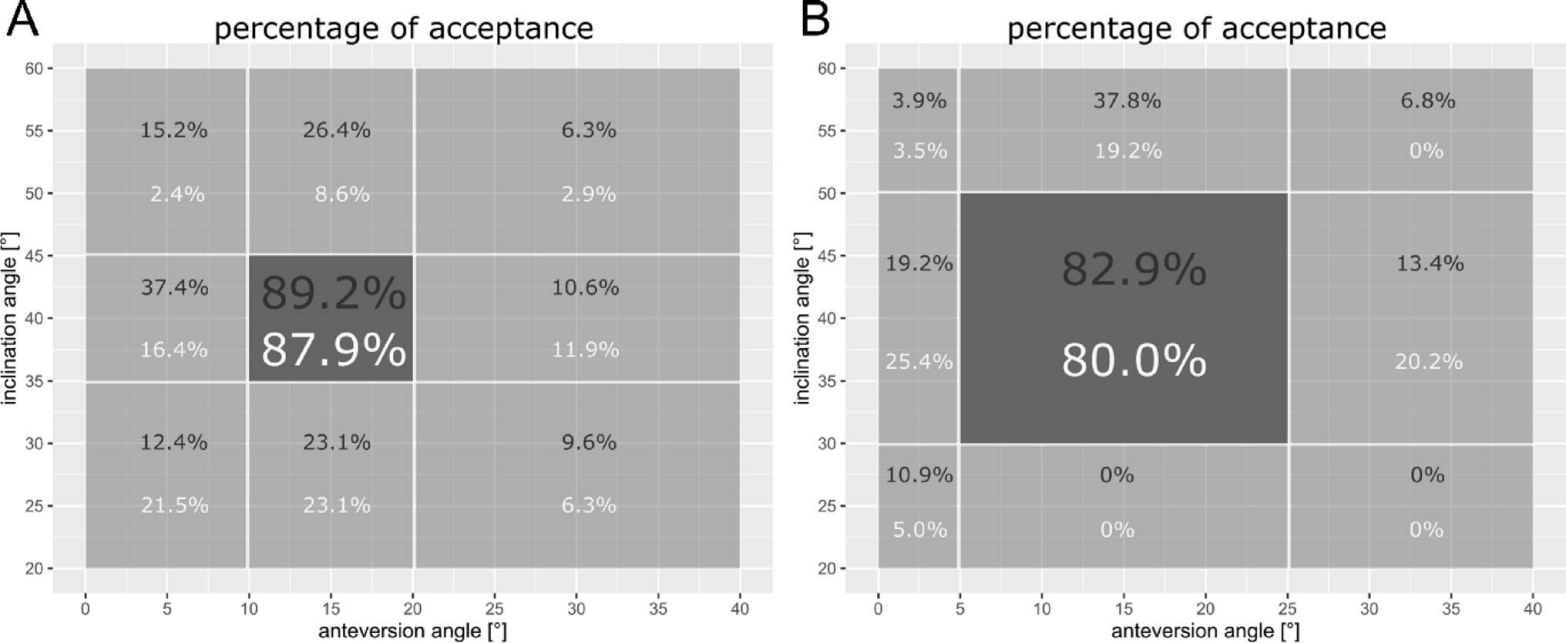
**A** Distribution of accepted cup orientations rated to be inside the institutional safe zone. **B** Distribution of accepted cup orientations rated to be inside the safe zone according to Lewinnek et al. Cup design 1 marked in black, and cup design 2 is marked in white. The correct cup orientation of design 1 was assessed correctly in 89.2% of all cases, whereas correct orientation of cup design 2 was assessed correctly in 87.9% of cases. For example, cup orientations with small inclination angles combined with correct anteversion angles were falsely accepted in 23.1% of cases for both cup designs according to the institutional safe zone definition

Figure 3A/B presents the comparative analysis of accurately assessing the positions of two distinct acetabular cup designs using two different safe zone definitions. The comprehensive evaluation of the assessments is detailed in Table 1. Overall, no statistically significant difference in the frequencies of correctly classified images based on both the institutional (*p* = 0.946) and Lewinnek’s safe zone (*p* = 0.643) was observed between the two cup designs (Fig. 3A/B).

**Table 1:** Comparison of the accuracy of assessment of the position of two distinct acetabular cup designs using two different safe zone definitions

	Sensitivity		Specificity	
	Cup design 1	Cup design 2	Cup design 1	Cup design 2
Institutional safe zone	89.2%	87.9%	84.0%	89.2%
Lewinnek’s safe zone	82.9%	80.0%	86.7%	88.6%

Sensitivity is defined as correctly identified cup orientation inside the two safe zones whereas specificity defines correctly identified cup orientation outside the safe zones. For example, a sensitivity of 89.2% means that 89.2% of all correct cup orientations were identified accordingly, leaving 10.8% of correct cup orientations falsely accounted to be outside the safe zone. Specificity of 84.0% means that 84.0% of all cup orientations outside the safe zone were correctly identified with 16.0% falsely estimated to be inside the safe zone

## Discussion

This study was set up to evaluate visually from fluoroscopy images the accuracy of estimation of acetabular cup orientation in THA, considering two different cup designs as well as the level of experience of the participants. Experience matters regarding correct identification of orientation within the defined safe zones. However, experience has no impact regarding the estimation of inclination and anteversion angles, as also demonstrated by Moreta et al. [22]. The cup design has only a marginal impact on the quality of estimation. Our findings also showed better sensitivity and equal specificity for the institutional safe zone, which was set narrower than Lewinnek’s safe zone. One possible reason for this finding is that the institutional safe zone is more commonly referenced and well-established in clinical practice, making it easier for surgeons to accurately assess cup orientations within these specific angles. In contrast, the wider Lewinnek range may be less frequently used, leading to more variability in assessments. Derived from our experimental study, an improved assessment of acceptable cup position may be achieved in clinical practice with the use of a narrower safe zone, compared to Lewinnek’s relatively wide definition. Furthermore, we agree that the difficulty in determining whether the angles fall within the acceptable range may be related to the familiarity with these thresholds, where less experienced raters may struggle more due to limited familiarity with the exact safe zone parameters. However, our study underscores that cup placement within target zones may be quite reliable just based on visual appreciation, without sophisticated tools for angle measurement. A condition would be obtaining a reliable projection of the fluoroscopy image, particularly implying correction of any tilt caused by a traction table.

As demonstrated in the results, estimation accuracy was highest for inclinations around 45° and anteversion angles between 0° and 15°. Since the Lewinnek safe zone includes a broader range of acceptable angles compared to the institutional safe zone, estimation at the edges of the Lewinnek range may be less reliable. This can lead to a lower percentage of correctly identified cup orientations within the safe zone. In clinical practice, this could result in more unnecessary corrections of cup orientations compared to the institutional safe zone, which showed a higher rate of correctly accepted orientations.

Known risk factors for cup malposition include obesity, a minimally invasive approach, and low surgical volume [23]. Placement of acetabular cups shows significant variation in inclination (21 to 73°) and anteversion (−17 to 43°) [23, 24]. Only 47% of the cups in a series of 1883 THA were within the optimal range of inclination (30 to 45°) and anteversion (5 to 25°) [23]. Bosker et al. [25] compared intraoperative estimations with postoperative radiographs, finding discrepancies in cup position despite using a cup impactor-positioner [25]. In total, 200 acetabular components were evaluated. Applying the limits according to Lewinneck (30–50° inclination and 5–25° anteversion), 70.5% of the cups were placed within this safe zone. Although, a statistically better performance of experienced surgeons was identified, whereas the difference to the residents was rather small. The study also demonstrated a tendency to underestimate both anteversion and inclination. The authors concluded that freehand placement is unreliable and that other techniques of cup positioning should be evaluated. In another clinical study, only 22% of the cups were placed within the safe zone when a specific mechanical acetabular alignment guide was used [8].

Different authors have suggested using intraoperative fluoroscopy to improve proper acetabular cup placement, particularly for the anterior approach [10–12]. However, data may be inconclusive and misleading as some studies have compared the anterior approach for THA in supine position assisted with fluoroscopy to the posterior approach in a lateral position [11, 12, 24, 26]. Beamer et al. evaluated intraoperative fluoroscopy for the anterolateral approach [27]. All patients were placed in a lateral position and the anterolateral approach was performed in most cases in a mixed series of primary and revision THA. However, other approaches were included as well without any further details given. Fluoroscopy increased accuracy of cup placement within the safe zone from 44 to 65%, improving both inclination and anteversion. Of interest, the subgroup analysis of primary THA failed to reach statistical significance.

Correct placement of the image intensifier is of paramount importance to assess acetabular cup placement. Patient positioning affects pelvic tilt and rotation, and fluoroscopy must compensate with corresponding adjustments to obtain a proper view [5, 13]. The pelvis may be oriented obliquely, particularly if a traction table is used, as it has no support under the ipsilateral buttock. An inlet view underestimates anteversion whereas it is overestimated towards an outlet view [14]. James et al. [15] obtained true ap pelvic views having the coccyx centered 2 to 2.5 cm cranially of the symphysis, as described as proper orientation [16]. However, the relative position of the coccyx varied widely on postoperative CT, from 3.5 cm below the symphysis to 2.5 cm above the symphysis, pelvic tilt ranging from 4° of flexion to 26° of extension [15]. Only 31% of the fluoroscopy views displayed a correct true ap view. Based on these findings, the authors recommended adjusting intraoperative fluoroscopy to match the size and shape of the obturator foramen to preoperative standing ap pelvic radiographs.

Even with all those aspects covered, such as a perfect fluoroscopic view compensating for any pelvic orientation, acetabular cup placement still depends on the surgeon’s ability to estimate inclination and anteversion. Different methods and formulas exist to calculate anteversion on postoperative radiographs, conversion tables also having been proposed [28–30]. However, most of these methods are not sufficiently evaluated, and there remains an issue with standardization of radiographic projection. Furthermore, most of these methods are applied on postoperative radiographs or computed tomography, but their intraoperative applicability use has not been evaluated yet. Thus, visual estimation is still used most commonly for intraoperative orientation with fluoroscopy.

One would assume that estimation of inclination is the easier task than estimating anteversion on plain antero-posterior radiographs. An anteversion of 0° can easily be estimated as the acetabular cup’s opening appears as a simple straight line. However, estimating anteversion with increasing angles appear to be more difficult since the minor axis of the ellipse or the shape of the ellipse itself is virtually converted into an angle and no simple angle can be estimated. Despite these considerations, the quality of estimation was similar for inclination and anteversion in our study. Our results are in line with an earlier study, which examined the ability of orthopaedic surgeons to visually estimate angles [31]. Correct estimation within 5° was achieved in only 64.6% of the time, and in 93.1% to within 10°. Angles of 31° or less were constantly overestimated. The number of years in practice or training was irrelevant to the performance.

The design of our study provided conditions for the participating orthopaedic surgeons which may not reflect the true reality encountered in the operation theatre. The image quality was excellent, and all the bony landmarks were easy to identify. No traction table interfered with imaging and the radiographs were adjusted for pelvic tilt and rotation. Although the rules were clear, we cannot exclude the use of a goniometer or other tools by any of the participants. There was no time limit set for the assessment. Thus, our results may underestimate out-of-range orientation of the cup in the clinical situation. Furthermore, only two different cup designs were tested. Cups with other shapes, such as true hemispherical designs or anatomically formed opening planes, may provide another visual impression and thus, influencing estimation of cup orientation.

Another definition of the safe zone would change sensitivity and specificity of being correctly identified upon visual estimation. However, changing the definitions of the safe zones would not change the main finding of our study, that inclination was overestimated for smaller angles with anteversion being increasingly overestimated with increasing anteversion angles.

It was the aim of the current study to evaluate the surgeon’s perception of inclination and anteversion on a plain radiography and our data do not allow to comment on the value of intraoperative fluoroscopy in general. Quality of cup orientation may be further improved by adding measuring tools and not solely relying on visual estimation of intraoperative fluoroscopy images. The combination of unrecognized or neglected intraoperative pelvic orientation, poor imaging technique and rather inadequate estimation of inclination and anteversion may result in a high rate of malpositioned acetabular cups.

## Conclusion

In this study, we investigated the accuracy of visually estimating cup inclination and anteversion using intraoperative fluoroscopy among orthopaedic surgeons with varying levels of experience. Our findings revealed that while experience and seniority did not significantly impact the estimation of cup orientation angles, sensitivity and specificity for identification of orientation within defined safe zone improved with increasing experience and seniority. Our study also highlighted the impact of anteversion angle on the estimation of inclination and vice versa, with marginal effects observed in both cases. Interestingly, we observed differences in the estimation of cup inclination between two distinct acetabular cup designs, with one design without lateral overhang and with a more pronounced pole flattening showing significantly better estimation than for the other. However, no significant difference was found regarding the estimation of anteversion between both designs. Moreover, our comparative analysis of safe zone definitions showed no significant difference in the frequencies of correctly classified images between the institutional and Lewinnek safe zones for the two cup designs. In conclusion, our experimental study sheds light on the complexities of estimation of acetabular cup orientation in THA and highlights the potential benefits of adopting a narrower safe zone in clinical settings. Further research is warranted to validate these findings and explore strategies for optimizing cup positioning techniques in THA.

## Data Availability

No datasets were generated or analysed during the current study.
